# Supplementary material for: Lens fluorescence and skin fluorescence in the Copenhagen Twin Cohort Eye Study: Covariates and heritability
Source: PLoS One. 2021 Sep 9;16(9):e0256975. doi: 10.1371/journal.pone.0256975 (PMC8428679; doi:10.1371/journal.pone.0256975)
Supplement: S1 File — (DOCX) [file pone.0256975.s001.docx]

## S1. Supplementary material 1

## Repeatability of fluorescence measurements

Among the three scans per subject, mean lens fluorescence of the lowest and highest measurements were 523 ng/ml [CI 501-547] and 582 ng/ml [CI 557- 609] respectively corresponding to -5.2 % [CI -9.2 % – (-)0.9 %] and +5.5 % [CI 1.0 % - 10.4 %] of mean lens fluorescence averaged from all three scans. There was no statistically significant difference between the means of the three measurements per subject as determined by three-group ANOVA analysis (p = 0.34, F = 1.2, DF = 2).

Among the three scans per subject, mean skin fluorescence of the lowest and highest measurements were 1.93 AU [1.87-1.99] and 2.17 AU [2.11-2.23] respectively corresponding to -5.9 % [CI -8.7 % – (-)2.8 %] and +5.9 % [CI 3.1 % - 8.9 %] of mean skin fluorescence averaged from all three scans per subject. There was no statistically significant difference between the means of the three measurements per subject as determined by three-group ANOVA analysis (p = 0.29, F = 1.2, DF = 2).
